# Computed Tomography Geometry of Extremely Undersized SAPIEN 3 Transcatheter Aortic Valves With Balloon Overfilling

**DOI:** 10.1016/j.jscai.2024.101937

**Published:** 2024-04-10

**Authors:** Richard Tanner, Parasuram Krishnamoorthy, Gilbert H.L. Tang, Stamatios Lerakis, Malcolm Anastasius, Amit Hooda, George D. Dangas, Samin K. Sharma, Annapoorna S. Kini, Sahil Khera

**Affiliations:** Mount Sinai Heart, Mount Sinai Hospital, New York, New York

**Keywords:** balloon-expandable, overfilling, transcatheter aortic valve replacement, valve expansion

Expansion of balloon-expandable valves in transcatheter aortic valve replacement (TAVR) beyond nominal volumes is feasible ex vivo.^1^ Balloon overfilling in SAPIEN 3/Ultra (S3) (Edwards Lifesciences) TAVR in undersized anatomies has had favorable short- to mid-term outcomes^2^, ^3^, ^4^; however, the degree of valve expansion achieved with balloon overfilling has not been systematically evaluated. Our study aimed to determine the computed tomography (CT)–derived geometry of S3 TAVR deployed with balloon overfilling in undersized anatomies and the associated impact on valve function.

From January 2019 to December 2022, 330 patients with tricuspid, severe native aortic stenosis underwent S3 TAVR undersized to the annular area. A total of 205 cases (62.1%) had extreme annular undersizing (EAU) that exceeded the manufacturer recommended upper limit (area >345 mm^2^ for 20 mm, >430 mm^2^ for 23 mm, >546 mm^2^ for 26 mm, and >683 mm^2^ for 29 mm), with 23 patients with EAU having a post-TAVR CT (23 mm, 10; 26 mm, 9; 29 mm, 4) to evaluate the S3 geometry. Patients underwent CT at follow-up to evaluate the frame expansion of S3 valves in undersized anatomies as part of a quality assurance measure. Balloon overfilling was defined as any volume added beyond nominal, based on annular and left ventricular outflow tract (LVOT) sizing and severity of annular/LVOT calcification. S3 geometry was evaluated using 3mensio imaging software (Pie Medical).^2^ Pre-CT and post-CT TAVR were performed on the same scanner, with analysis being performed by the same reviewer. Eccentricity index was defined as the minimum diameter of the valve divided by the maximum diameter. The valve expansion index was calculated by dividing the cross-sectional area by the nominal area for each valve size. Follow-up was 100% complete, and clinical and echocardiographic outcomes, per Valve Academic Research Consortium-3, were reported. Our study received institutional review board approval, and the need for patient consent was waived.

**Figure 1:**
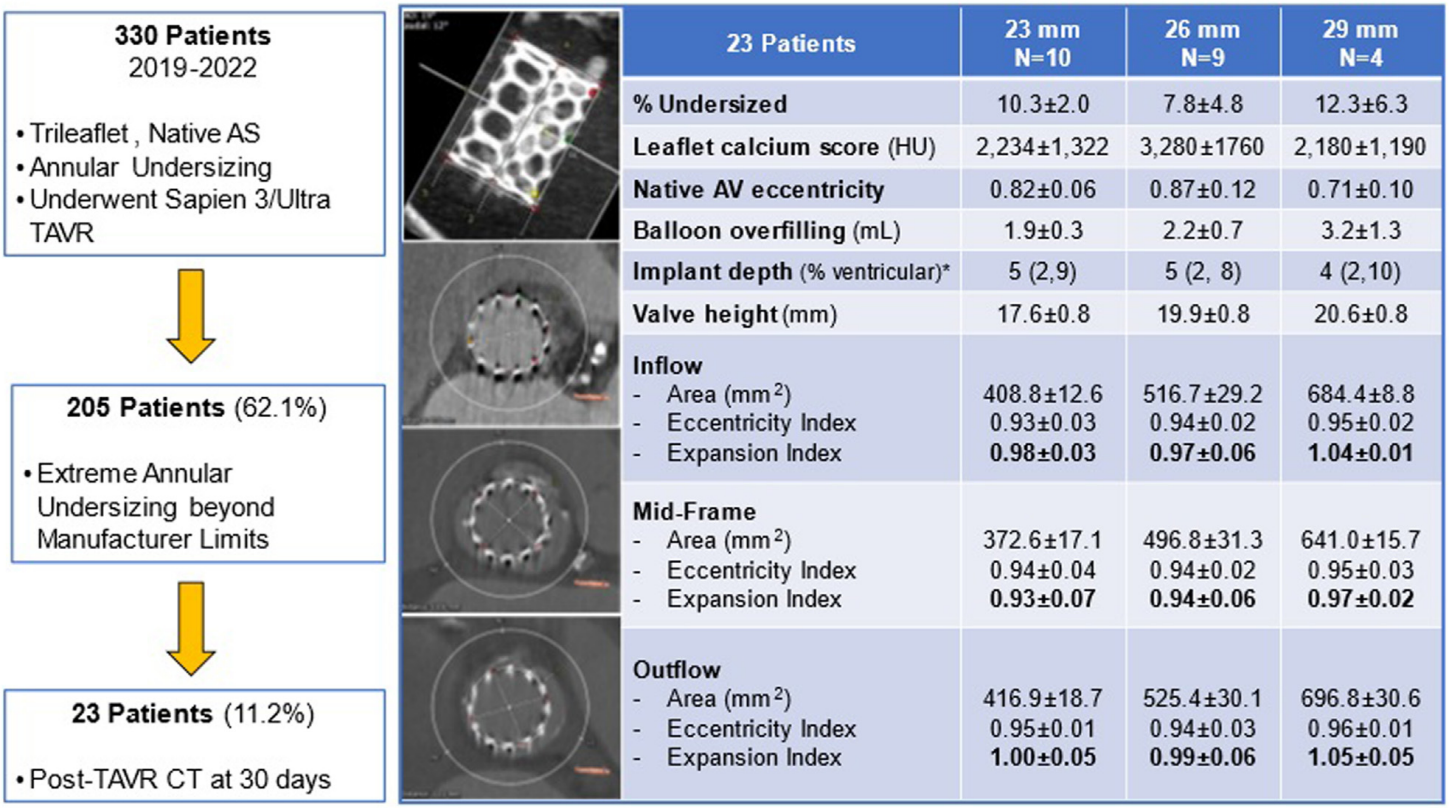
**Summary of our post-TAVR computed tomography (CT) study on balloon overfilling in SAPIEN 3 TAVR in extremely undersized anatomies.** AS, aortic stenosis; AV, aortic valve; HU, Hounsfield units; TAVR, transcatheter aortic valve replacement. ∗Represented by median and interquartile range.

Of the 23 patients with post-TAVR CT, the mean age was 76 ± 7.2 years, the Society of Thoracic Surgeons risk score was 3.1% ± 1.8%, with 17.4% being female. On semiquantitative analysis, no patient had moderate or severe annular or LVOT calcification. Annular undersizing for each valve was 10.3% ± 2.0% for 23 mm, 7.8% ± 4.8% for 26 mm, and 12.3% ± 6.3% for 29 mm. Mean volumes added to 23 mm, 26 mm, and 29 mm S3 valves were 1.9 ± 0.3 mL, 2.2 ± 0.7 mL, and 3.2 ± 1.3 mL, respectively. All patients underwent successful transfemoral TAVR with conscious sedation. Postdilatation occurred in 2 patients (8.7%), with the same volume being used in one case and an additional 1 mL in the other. Post-TAVR CT dimensions and geometric indices of each valve size at the inflow, midframe, and outflow are shown in Figure 1. No valve achieved an expansion index of >1 at the midframe portion, ie, greater than the respective valve’s nominal valve area, but 29 mm S3 had an expansion index of >1 at inflow and outflow. One patient with 26 mm S3 had hypoattenuating leaflet thickening on 30-day CT that resolved after anticoagulation.

No periprocedural complications occurred. At 30 days, mean gradients were 14.40 ± 5.15 mm Hg for 23 mm, 11.1 ± 4.0 mm Hg for 26 mm, and 8.0 ± 2.2 mm Hg for 29 mm S3, respectively. Paravalvular leak (PVL) was mild in 20.0% of 23 mm, 22.2%% in 26 mm, and 0% in 29 mm S3, respectively, with no moderate/severe PVL. No patient had mild or greater transvalvular aortic regurgitation.

We have previously reported EAU’s feasibility and favorable outcomes with S3 TAVR.^3^, ^4^, ^5^ This study showed that despite EAU and balloon overfilling, the valve expansion index was <1 in all S3 valves at the midframe, and the final dimensions, except the inflow and outflow of the 29 mm S3, were smaller than the nominal areas. A greater expansion of the 29 mm S3 may reflect the higher volume of fluid added and the percentage of undersizing. Despite this, 30-day echocardiography showed no worsening PVL or transvalvular aortic regurgitation. A recent large-scale CT study showed that underexpanded valves predicted hypoattenuating leaflet thickening and all-cause mortality at 1 year.^6^ Our CT study suggests that balloon overfilling in EAU did not significantly overexpand S3 valves. Given that the 29 mm S3 has the tallest frame, it may be more amenable to overexpansion at the inflow and outflow, as shown in our study. We hypothesize that a more optimal valve expansion can be achieved with S3 TAVR, when undersizing the valve for the annular anatomy, adding volume to the delivery balloon during deployment, or postdilatation to achieve a more complete expansion.

Our single-center cohort is limited by small sample size and lack of core laboratory evaluation, and long-term follow-up is required before drawing conclusions about durable clinical and echocardiographic outcomes with our S3 implant strategy. However, to our knowledge, this is the first study to assess post-TAVR CT-derived geometry of overfilled balloon-expandable valves, and the fact that no valve became overexpanded may prompt further investigation into the current balloon-expandable TAVR sizing strategy to achieve optimal valve expansion.

In the future, the SAPIEN X4 system may facilitate more accurate expansion as deployment diameters in 0.5 mm increments will be available.
